# Asiatic Acid Exhibits Anti-inflammatory and Antioxidant Activities against Lipopolysaccharide and d-Galactosamine-Induced Fulminant Hepatic Failure

**DOI:** 10.3389/fimmu.2017.00785

**Published:** 2017-07-07

**Authors:** Hongming Lv, Zhimin Qi, Sisi Wang, Haihua Feng, Xuming Deng, Xinxin Ci

**Affiliations:** ^1^Department of Translational Medicine, The First Hospital of Jilin University, Changchun, China; ^2^Key Laboratory of Zoonosis, Ministry of Education, College of Veterinary Medicine, Jilin University, Changchun, China

**Keywords:** asiatic acid, inflammation, oxidative stress, fulminant hepatic failure, AMPK/Nrf2, PDCD4

## Abstract

Inflammation and oxidative stress are essential for the pathogenesis of fulminant hepatic failure (FHF). Asiatic acid (AA), which is a pentacyclic triterpene that widely occurs in various vegetables and fruits, has been reported to possess antioxidant and anti-inflammatory properties. In this study, we investigated the protective effects of AA against lipopolysaccharide (LPS) and d-galactosamine (GalN)-induced FHF and the underlying molecular mechanisms. Our findings suggested that AA treatment effectively protected against LPS/d-GalN-induced FHF by lessening the lethality; decreasing the alanine transaminase and aspartate aminotransferase levels, interleukin (IL)-1β, IL-6, and tumor necrosis factor-α production, malondialdehyde formation, myeloperoxidase level and reactive oxygen species generation (i.e., H_2_O_2_, NO, and $O_{2}^{-}$), and increasing the glutathione and superoxide dismutase contents. Moreover, AA treatment significantly inhibited mitogen-activated protein kinase (MAPK) and nuclear factor-kappa B (NF-κB) signaling pathway activation *via* the partial induction of programmed cell death 4 (PDCD4) protein expressions, which are involved in inflammatory responses. Furthermore, AA treatment dramatically induced the expression of the glutamate-cysteine ligase modifier subunit, the glutamate-cysteine ligase catalytic subunit, heme oxygenase-1, and NAD (P) H: quinoneoxidoreductase 1 (NQO1), which are largely dependent on activation of the nuclear factor-erythroid 2-related factor 2 (Nrf2) through the induction of AMP-activated protein kinase (AMPK) and glycogen synthase kinase-3β (GSK3β) phosphorylation. Accordingly, AA exhibited protective roles against LPS/d-GalN-induced FHF by inhibiting oxidative stress and inflammation. The underlying mechanism may be associated with the inhibition of MAPK and NF-κB activation *via* the partial induction of PDCD4 and upregulation of Nrf2 in an AMPK/GSK3β pathway activation-dependent manner.

## Introduction

The liver is a vital organ that is vulnerable to multiple factors, including alcohol, chemical substances, oxidative products, and the hepatitis viruses, which lead to hepatic failure (1). Fulminant hepatic failure (FHF) is a life-threatening and fatal clinical syndrome that is associated with a poor prognosis and high mortality (2). The lipopolysaccharide (LPS) and d-galactosamine (GalN)-induced animal model of FHF is strongly relevant to human liver failure and has been widely used to investigate the mechanisms and potential therapeutic drugs for clinical FHF (3). Increasing evidence has shown that oxidative stress and inflammatory responses are two important pathogenic factors that contribute to LPS/d-GalN-induced FHF (4). Consequently, inhibiting inflammation and/or oxidative stress may be potential prevention measures for the development of FHF.

**Figure 1:**
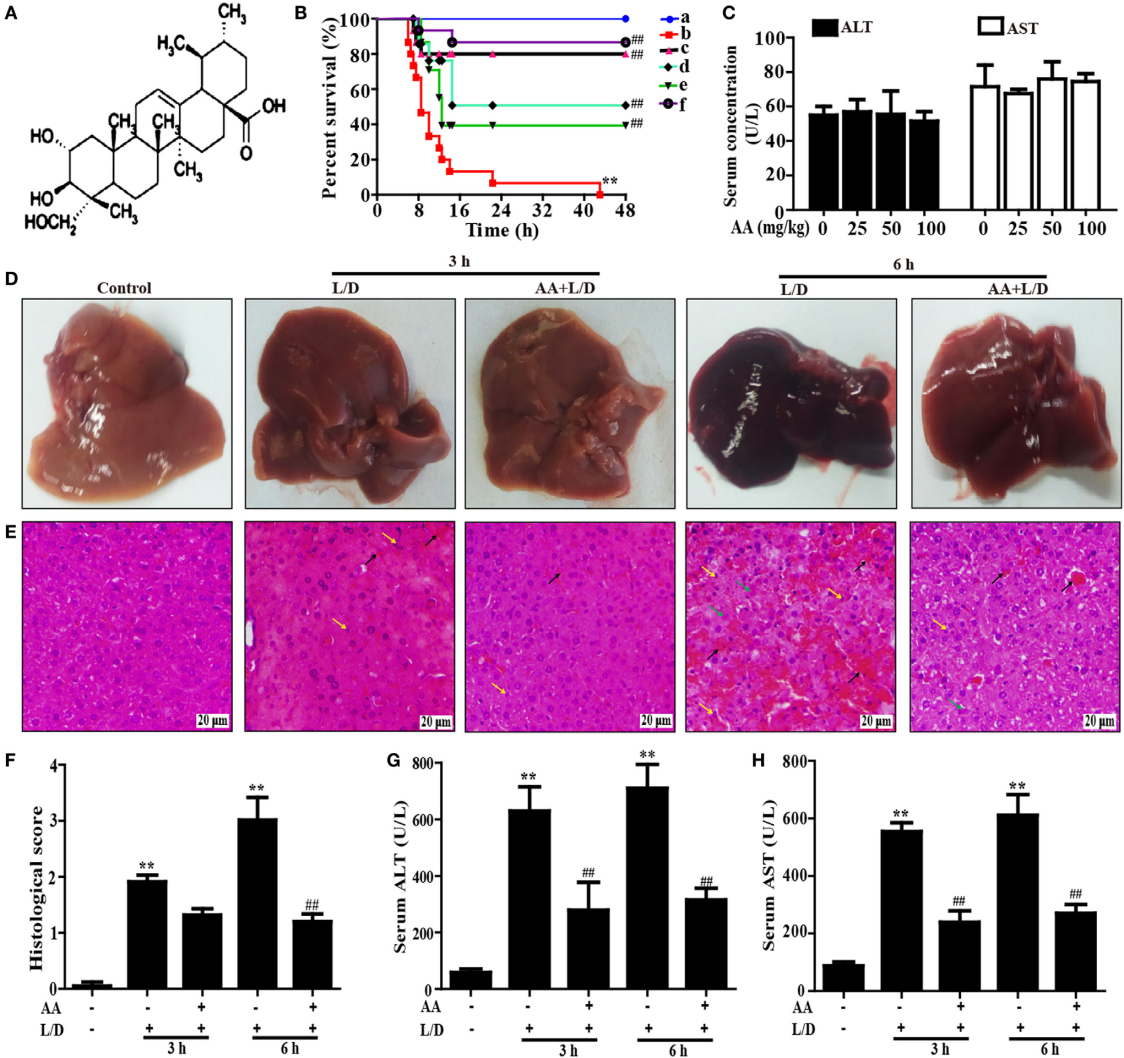
Protective effect of AA treatment on lipopolysaccharide (LPS)/d-galactosamine (GalN)-induced fulminant hepatic failure. **(A)** The chemical structure of asiatic acid (AA). **(B)** AA (6.25, 12.5, or 25 mg/kg) or hemin (30 mg/kg) was administered intraperitoneally to mice for twice at a 12-h (interval for 12 h), followed by subjected treatment with LPS (30 µg/kg) and d-GalN (600 mg/kg), which is abbreviated as L/D. The survival rates of the mice were observed within 48 h after L/D exposure. (a) Control and AA group; (b) L/D group; (c) AA (25 mg/kg) + L/D; (d) AA (12.5 mg/kg) + L/D; (e) AA (6.25 mg/kg) + L/D; (f) hemin (30 mg/kg) + L/D. **(C)** Mice only were given an intraperitoneal injection of AA (0, 25, 50, and 100 mg/kg) and we harvested serum for the analysis of alanine transaminase (ALT) and aspartate aminotransferase (AST) levels at 6 h. **(D)** Livers (*n* = 5) from each experimental group were processed for gross examination of liver at 3 or 6 h after the L/D challenge. **(E)** Representative histological sections of the livers were stained with hematoxylin and eosin (H&E)-stained (magnification 400×, black arrows: hemorrhage; green arrows: necrotic area; yellow arrows: inflammatory cell infiltration). **(F)** The stained sections were graded using a four-point scale from 0 to 3, with 0, 1, 2, and 3 representing no damage, mild damage, moderate damage, and severe damage, respectively. Additionally, sera were collected from the mice after exposure to L/D for 3 and 6 h for measurement of the ALT and AST levels. **(G,H)** Effects of AA on the serum ALT and AST levels. Similar results were obtained from three independent experiments. All data are presented as means ± SEM (*n* = 5 in each group). *p < 0.05 and **p < 0.01 vs. control group; ^#^*p* < 0.05 and ^##^*p* < 0.01 vs. L/D group.

Previous abundant reports have shown that LPS/GalN-induced FHF, which is a type of toxin-induced liver injury, is dependent upon macrophage-derived inflammatory cytokines, such as interleukin (IL)-1β, IL-6, and tumor necrosis factor (TNF)-α. These cytokines are regulated by the activation of multiple signaling pathways, including toll-like receptor 4-mediated mitogen-activated protein kinase (MAPK); this pathway includes the c-Jun NH2-terminal kinase (JNK), extracellular signal-regulated kinase (ERK), p38, and nuclear factor-kappa B (NF-κB), which comprise the p50/p65 and the inhibitor of κB (IκB) protein signaling pathways (5, 6). Importantly, the tumor suppressor programmed cell death-4 (PDCD4) gene, which was initially regarded as an upregulated gene during apoptosis, is universally expressed in normal tissues, with the highest levels found in the liver (7). Additionally, PDCD4 can mediate inflammatory responses that play essential roles in the amelioration of LPS/d-GalN-induced acute liver injury *via* inhibition of MAPK and NF-κB pathway activation (8). More intriguingly, apart from inflammatory responses, the excessive accumulation of reactive oxygen species (ROS) is recognized to a possible mechanism of d-GalN/LPS-induced FHF (9). ROS overproduction not only directly triggers oxidative damage but also activates the MAPK and NF-κB signaling pathways, resulting in inflammatory responses that further stimulate inflammatory injury (10, 11). Furthermore, nuclear factor erythroid 2-related factor 2 (Nrf2), which is a key transcription factor that is required to ameliorate various oxidative stress- and inflammation-associated diseases, regulates the expression of various antioxidant genes, including heme oxygenase-1 (HO-1), NAD (P) H: quinoneoxidoreductase 1 (NQO1), and the glutamate-cysteine ligase modifier (GCLM) and glutamate-cysteine ligase catalytic (GCLC) subunit (12, 13). Previously, several reports implied that Nrf2 activation played vital roles in the pathogenesis of liver injury both *in vitro* and *in vivo* (14). To date, Nrf2 activation has been reported to be regulated by the AMP-activated protein kinase (AMPK) and subsequent inactivation of glycogen synthase kinase-3β (GSK-3β) (15, 16). Accumulating evidence has shown that various natural products, including phenols, coumarins, triterpenoid, flavonoids, and alkaloids protect against hepatic diseases through activation of the Nrf2 pathway (17, 18). Asiatic acid (AA) (Figure 1A) is a pentacyclic triterpene that widely occurs in many vegetables and fruits and has been reported to possess a variety of biological activities, including antioxidant and anti-inflammatory properties (19, 20). AA has been reported to reduce the pulmonary inflammation induced by cigarette smoking and inhibit liver fibrosis by blocking TGF-beta/Smad signaling *in vivo* and *in vitro* (21, 22). In this study, we explored whether AA attenuation of LPS/d-GalN-induced hepatotoxicity was associated with the induction of AMPK/GSK3β-Nrf2 and PDCD4. Our results indicated that AA treatment attenuated LPS/d-GalN-induced hepatotoxicity and inhibited inflammatory responses and oxidative stress, which possibly involved in the suppression of MAPK and NF-κB activation *via* the partial induction of PDCD4 and upregulation of Nrf2 in an AMPK/GSK3β pathway activation-dependent manner.

## Materials and Methods

### Reagents and Chemical

Asiatic acid, purity >98%, was provided by the Chengdu Herbpurify Co., Ltd (Chengdu, China). LPS (*Escherichia coli* 055:B5), GalN, and dimethyl sulfoxide were purchased from Sigma-Aldrich (St. Louis, MO, USA). Inhibitors of AMPK (Compound C) was offered by Sigma-Aldrich (St. Louis, MO, USA). Fetal bovine serum, Dulbecco’s modified Eagle’s medium, penicillin, and streptomycin were acquired from Invitrogen-Gibco (Grand Island, NY, USA). Antibodies against Nrf2, GCLC, GCLM, HO-1, NQO1, P-AMPK, AMPK, P-PI3K, PI3K, P-AKT, AKT, P-ERK, ERK, PDCD4, P-P65, P65, IκBα, P-IκBα, and β-actin were obtained from Cell Signaling (Boston, MA, USA) or Abcam (Cambridge, MA, USA). Additionally, $O_{2}^{-}$, H_2_O_2_, NO, glutathione (GSH), superoxide dismutase (SOD), malondialdehyde (MDA), myeloperoxidase (MPO), and ROS test kits were supplied by Nanjing Jiancheng Bioengineering Institute (Nanjing, China). All other chemicals were offered by Sigma-Aldrich (St. Louis, MO, USA), if not otherwise indicated.

### Animals

Female BALB/c mice (6–8 weeks), weighing approximately 18–20 g, were purchased from Liaoning Changsheng Technology Industrial Co., LTD (Certificate SCXK2010-0001; Liaoning, China). All animals were housed in a room with temperature at 24 ± 1°C, a 12 h light–dark cycle and relative humidity about 40–80%. Animals were allowed free access to tap water and normal food after feeding several days. All animal experiments were performed according to the guide for the Care and Use of Laboratory Animals, which was published by the US National Institute of Health. This study was reviewed and approved by the Animal Welfare and Research Ethics Committee at Jilin University.

### Experimental Protocol

Mice were randomly divided into seven groups: control (PBS) group, AA only (25 mg/kg) group, LPS/d-GalN (L/D, 30 µg/kg and 600 mg/kg) group, and AA (6.25, 12.5, or 25 mg/kg) + (L/D) group and hemin (a HO-1 inducer as a positive group, 30 mg/kg) + (L/D) group, were administered intraperitoneally. In brief, AA (6.25, 12.5, or 25 mg/kg) or hemin (30 mg/kg) was administered intraperitoneally to mice for twice at a 12-h (interval for 12 h), followed by subjected treatment with LPS (30 µg/kg) and d-GalN (600 mg/kg), which is abbreviated as L/D. The survival rates of mice were observed for 48 h after L/D challenge. For other assays, after L/D administration for 3 and 6 h, the animals were euthanized. Subsequently, liver tissues and serum were harvested and used for hematoxylin and eosin (H&E) staining, Western blot assay, and enzyme-linked immunosorbent assay (ELISA).

### Histopathological Evaluation

Liver tissues were immersed in normal 10% neutral buffered formalin and fixed for 48 h, dehydrated in a series of graded ethanol, embedded in paraffin wax, and cut into 5-µm-thick sections. The paraffin-embedded sections were stained with hematoxylin and eosin (H&E) for pathological analysis under a light microscope. The histological changes were evaluated by a point-counting method for severity of hepatic injury using an ordinal scales in accordance with the methods as previous described (23). Briefly, H&E-stained sections were evaluated at 400× magnification by a point-counting method for severity of hepatic injury using an ordinal scale as follows; grade 0: minimal or no evidence of injury, grade 1: mild injury consisting in cytoplasmic vacuolation and focal nuclear pyknosis, grade 2: moderate to severe injury with extensive nuclear pyknosis, cytoplasmic hypereosinophilia, and loss of intercellular borders, and grade 3: severe necrosis with disintegration of hepatic cords, hemorrhage, and neutrophil infiltration.

### Biochemical Indexes Assay

All mice were euthanized at 3 or 6 h after L/D treatment, liver and blood were collected for biochemical analysis. alanine transaminase (ALT) and aspartate aminotransferase (AST) levels in serum were measured using the corresponding detection kits. In addition, mice liver tissues were homogenized and dissolved in extraction buffer to analyze the MPO, GSH, MDA, and SOD levels according to the manufacturer’s instructions. All results were normalized by the total protein concentration in each sample. For other assays, all mice were sacrificed at 3 or 6 h after L/D treatment, liver tissues were collected for measurement of $O_{2}^{-}$, NO, H_2_O_2_, and ROS generation. Mice liver tissues were homogenized and dissolved in extraction buffer to analyze the $O_{2}^{-}$, NO, H_2_O_2_, and ROS levels in accordance to the manufacturer’s instructions.

### ELISA Assay

Blood was obtained from each sample *in vivo*, centrifuged, collected serum for measurement of the TNF-α, IL-6, and IL-1β secretion using an ELISA kit as the manufacturer’s instructions (BioLegend, Inc., CA, USA), respectively. The optical density from each well was detected at 450 nm.

### Western Blot Analysis

Liver tissues were collected 3 or 6 h after L/D challenge. Total protein was extracted from the liver tissues using a protein extract kit according to the manufacturer’s protocol. Protein concentrations were tested by the BCA method. Equal amounts of proteins (20 µg) were separated by a 10% SDS-polyacrylamide gel and transferred onto a polyvinylidene difluoride membrane. The membrane was blocked with 5% (w/v) non-fat milk for 2 h. Then, the membrane was incubated with primary antibody and secondary antibody. Finally, the membranes were visualized by the ECL Western blotting detection system in accordance with the manufacturer’s instruction and band intensities were quantified using Image J gel analysis software. All experiments were performed in triplicate.

### CRISPR/Cas9 Knockout of Nrf2 Gene (24)

HepG2 cells were cultured in 12-well plates at the density of 3 × 10^5^ cells/well for 24 h. The plasmids of expressing Cas9 with Nrf2-sgRNA and puromycin resistant gene were co-transfected into HepG2 cells using Viafect transfection reagent (Promega). At 48 h after transfection, cells were added puromycin at a concentration of 2 µg/mL and harvested for immunoblotting analysis with Nrf2 antibody. After 7 days, cells were cultured in a 96-well plates (1 cell/well).

### Statistical Analysis

All data referenced above were expressed as the means ± SEM and analyzed using SPSS19.0 (IBM). Comparisons between experimental groups were conducted using one-way ANOVA, whereas multiple comparisons were made using the LSD method. Statistical significance was defined as *p* < 0.05 or *p* < 0.01.

## Results

### AA Treatment Protected Mice against LPS/d-GalN (L/D)-Induced FHF

To investigate whether AA could protect against L/D-induced liver injury in mice, the survival rates of the mice were observed for 48 h after L/D exposure. As shown in Figure 1B, the mice died 7 h after L/D injection, and the survival rate was 0% at 48 h. Conversely, pretreatment with AA (25 mg/kg) effectively increased the survival rate up to 80% and no significant difference compared with hemin (a HO-1 inducer) group, a positive group. In addition, because serum AST and ALT levels were used as well-established marker of hepatic injury (25), the serum ALT and AST levels were measured. Our results indicated that no changes in serum levels of ALT and AST were induced by alone AA at various dosages ranging from 0 to 100 mg/kg, indicating that AA did not exhibit hepatotoxicity (Figure 1C). Meanwhile, we found that the serum ALT and AST levels were significantly increased by L/D administration compared with the control group (*p* < 0.01). However, this increase was markedly reduced by AA pretreatment, suggesting that AA treatment efficiently protected against L/D-induced FHF (Figures 1G,H) (*p* < 0.01). Gross and histological examinations of liver tissues were used to evaluate the protective effects of AA on L/D-induced FHF. As illustrated in Figure 1D, gross examination of the livers displayed apparent congestion and hemorrhage in the L/D-induced group mice 6 h after challenge, indicating severe liver injury. Similarly, histological analysis of the mouse liver sections in the L/D group showed obviously disturbed architecture, such as hepatocyte necrosis, hemorrhage, and neutrophil infiltration. However, the L/D-induced liver alterations were effectively relieved by AA treatment based on the liver injury scores (Figures 1E,F).

### AA Treatment Reduced Inflammatory Responses in Mice with L/D-Induced FHF

**Figure 2:**
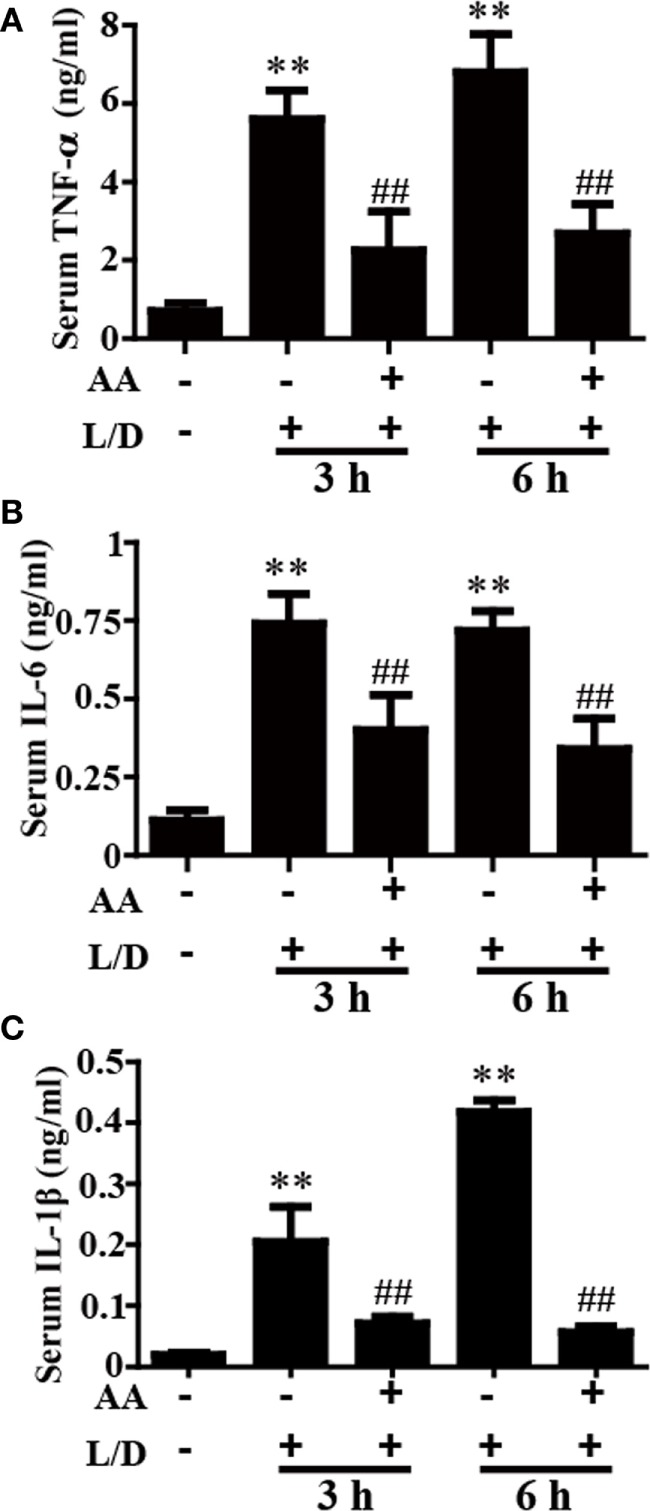
Effect of asiatic acid (AA) treatment on inflammatory cytokines in L/D-induced fulminant hepatic failure. AA (25 mg/kg) was administered intraperitoneally to mice 1 h before L/D pretreatment. **(A–C)** Effects of AA on L/D-induced serum tumor necrosis factor (TNF)-α, interleukin (IL)-6, and IL-1β generation. Similar results were obtained from three independent experiments. All data are presented as means ± SEM (*n* = 5 in each group). *p < 0.05 and **p < 0.01 vs. control group; ^#^*p* < 0.05 and ^##^*p* < 0.01 vs. L/D group.

The inflammatory cytokines TNF-α, IL-6, and IL-1β play vital roles in liver injury. To further explore the anti-inflammatory effects of AA, the effects of AA on TNF-α, IL-6, and IL-1β generation in the sera was measured by ELISA. As shown in Figures 2A–C, L/D effectively increased the secretion of TNF-α, IL-6, and IL-1β (*p* < 0.01) in the sera, whereas AA treatment inhibited the induction of inflammatory cytokine production by L/D (*p* < 0.01).

### AA Treatment Inhibited the MAPK and NF-κB Activation and Induced PDCD4 Protein Expression in Mice with L/D-Induced FHF

**Figure 3:**
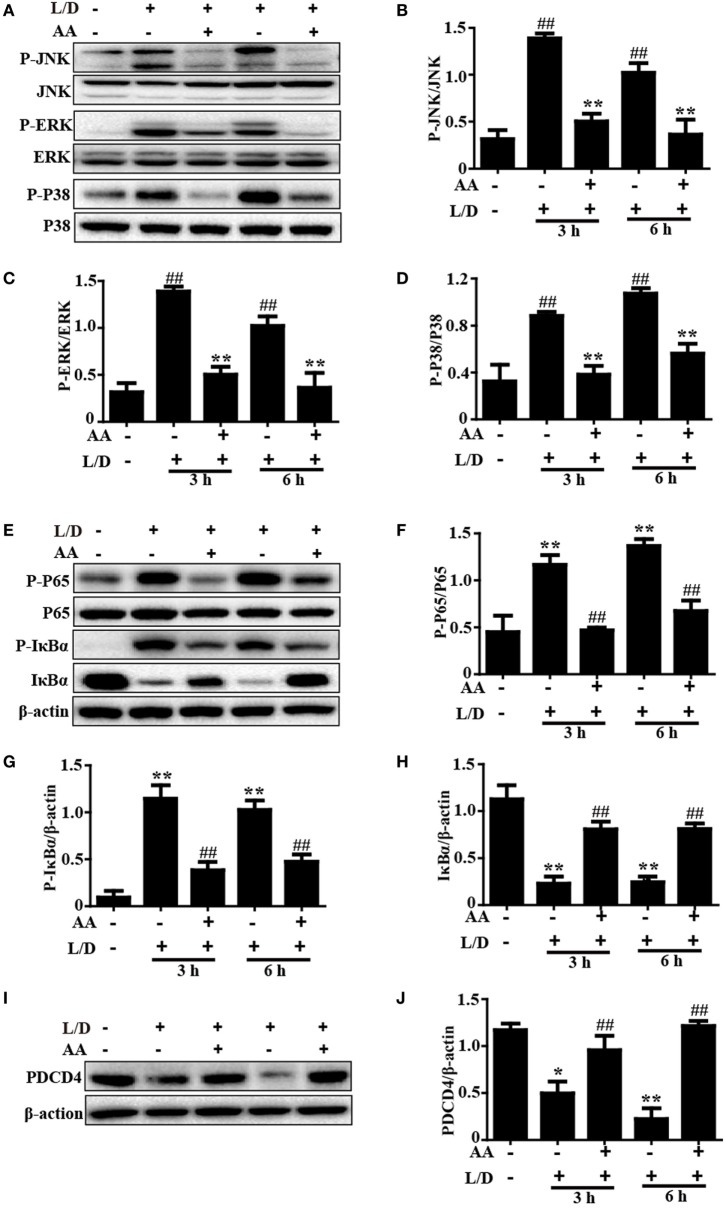
Inhibitory effects of asiatic acid (AA) treatment on NF-κB, mitogen-activated protein kinase, and PDCD4 in L/D-induced fulminant hepatic failure. Liver tissues were collected from the mice 3 or 6 h after L/D challenge and analyzed by Western blotting. **(A,E,I)** Effects of AA on P-JNK, P-extracellular signal-regulated kinase (ERK), P-P38, P-P65, P-IκBα, IκBα, and PDCD4 expression were measured by Western blotting. **(B–D,F–H,J)** Quantification of relative protein expression was performed by densitometric analysis. Similar results were obtained from three independent experiments. All data are presented as means ± SEM (*n* = 5 in each group). *p < 0.05 and **p < 0.01 vs. control group; ^#^*p* < 0.05 and ^##^*p* < 0.01 vs. L/D group.

Because the NF-κB and MAPK signaling pathways have been reported to be inflammatory pathways that play imperative roles in mice with L/D-induced FHF, we evaluated the effects of AA treatment on the L/D-induced activation of the NF-κB signaling pathway. As presented in Figures 3A–H, AA treatment remarkably inhibited P65, JNK, ERK, and P38 phosphorylation (*p* < 0.01) and blocked IκBα phosphorylation and degradation compared to the L/D-challenged group, suggesting the inhibition of inflammatory responses by AA might be partially responsible for blocking the activation of the NF-κB and MAPK signaling pathways.

Importantly, PDCD4 negatively or positively mediates inflammatory responses, although this topic is still controversial. Wang et al. (26) discovered that PDCD4 deficiency aggravated colitis *via* promoting the IL-6/STAT3 pathway in mice. However, Schmid et al. (27) maintained that inflammation could induce the loss of PDCD4, and Wang et al. (8) indicated that PDCD4 upregulation inhibited inflammatory responses by inhibiting NF-κB and MAPK signaling pathway activation in an LPS/d-GalN-induced mouse model. Therefore, PDCD4 may be closely associated with inflammatory responses. Indeed, we found that the PDCD4 protein expression level was dramatically reduced 3 and 6 h after L/D injection (*p* < 0.05 and *p* < 0.01, respectively) in our studies. Conversely, AA pretreatment effectively restored PDCD4 protein expression (Figures 3I,J), indicating that inhibition of the inflammatory responses induced by L/D challenge by AA might be partially attributed to the increased PDCD4 protein expression (*p* < 0.01).

### AA Treatment Attenuated L/D-Triggered Oxidative Damage in Mice with FHF

**Figure 4:**
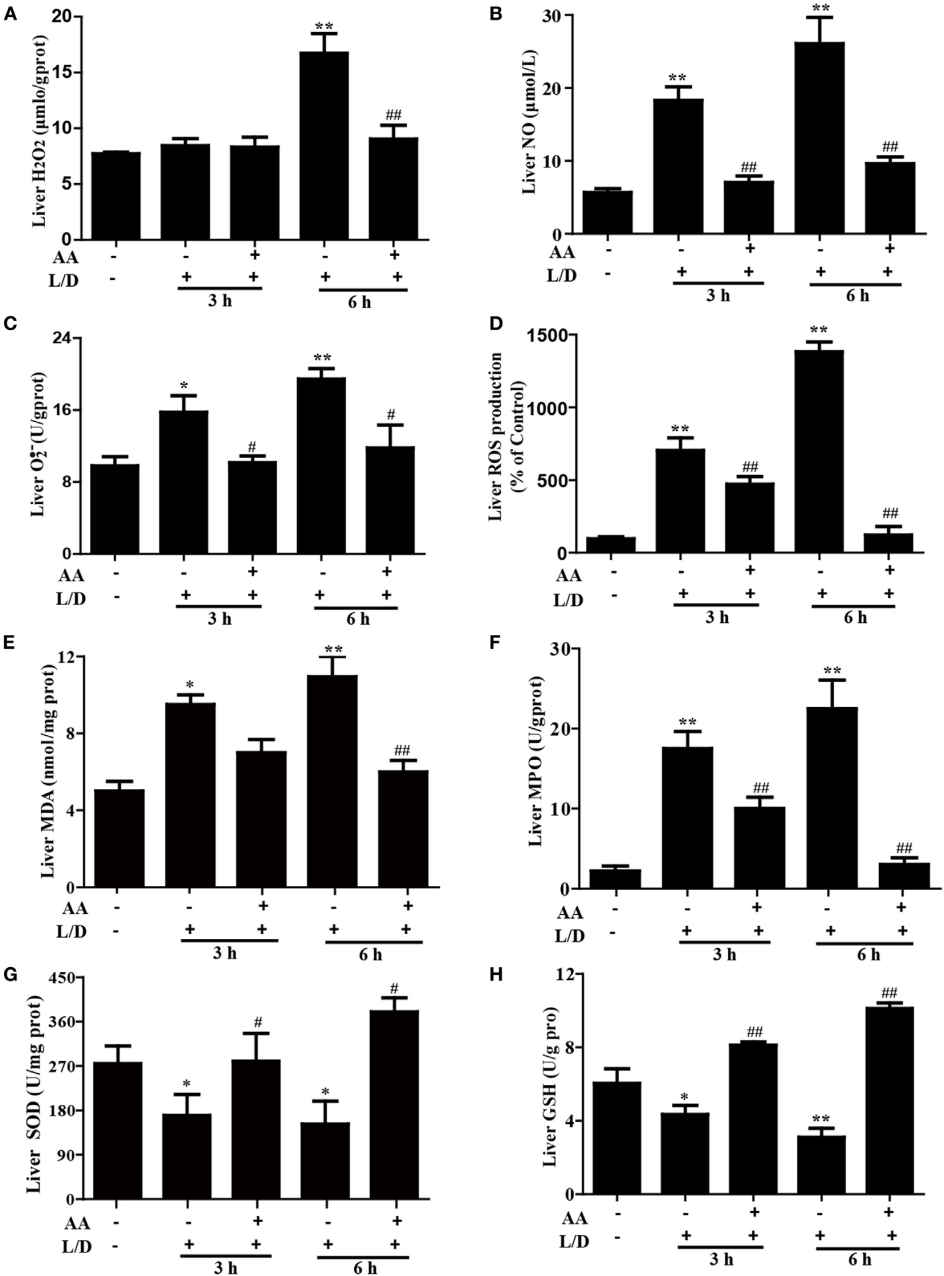
Effects of asiatic acid (AA) treatment on the levels of oxidative markers in L/D-induced fulminant hepatic failure. Liver tissues were collected from the mice 3 or 6 h after L/D challenge for measurement of the generation of H_2_O_2_, NO, $O_{2}^{-}$, and reactive oxygen species (ROS), formation of malondialdehyde (MDA) and myeloperoxidase (MPO), and the superoxide dismutase (SOD) and glutathione (GSH) activities. **(A–D)** Effects of AA on liver H_2_O_2_, NO, ${\text{O}}_{2}^{-}$, and ROS production. **(E,F)** Effects of AA on the liver MDA and MPO levels. **(G,H)** Effects of AA on the liver SOD and GSH activities. Similar results were obtained from three independent experiments. All data are presented as means ± SEM (*n* = 5 in each group). *p < 0.05 and **p < 0.01 vs. control group; ^#^*p* < 0.05 and ^##^*p* < 0.01 vs. L/D group.

Because oxidative damage is a major factor in mice with L/D-induced FHF, we determined whether AA pretreatment attenuated the L/D-triggered oxidative stress. As shown in Figure 4, L/D not only evidently increased MDA formation (*p* < 0.05 or *p* < 0.01), the MPO level (*p* < 0.01), and ROS generation (i.e., H_2_O_2_, NO, and $O_{2}^{-}$ production) (*p* < 0.05 or *p* < 0.01) but also obviously decreased the SOD (*p* < 0.05) and GSH (*p* < 0.01) contents in the livers of the mice. In contrast, AA pretreatment dramatically inhibited these effects induced by L/D (*p* < 0.05 or *p* < 0.01). These observations indicated that AA treatment ameliorated hepatic injury by partially lessening L/D-triggered oxidative stress in mice.

### AA Treatment Upregulated the Nrf2 and AMPK/GSK3β Signaling Pathways in Mice with L/D-Induced FHF

**Figure 5:**
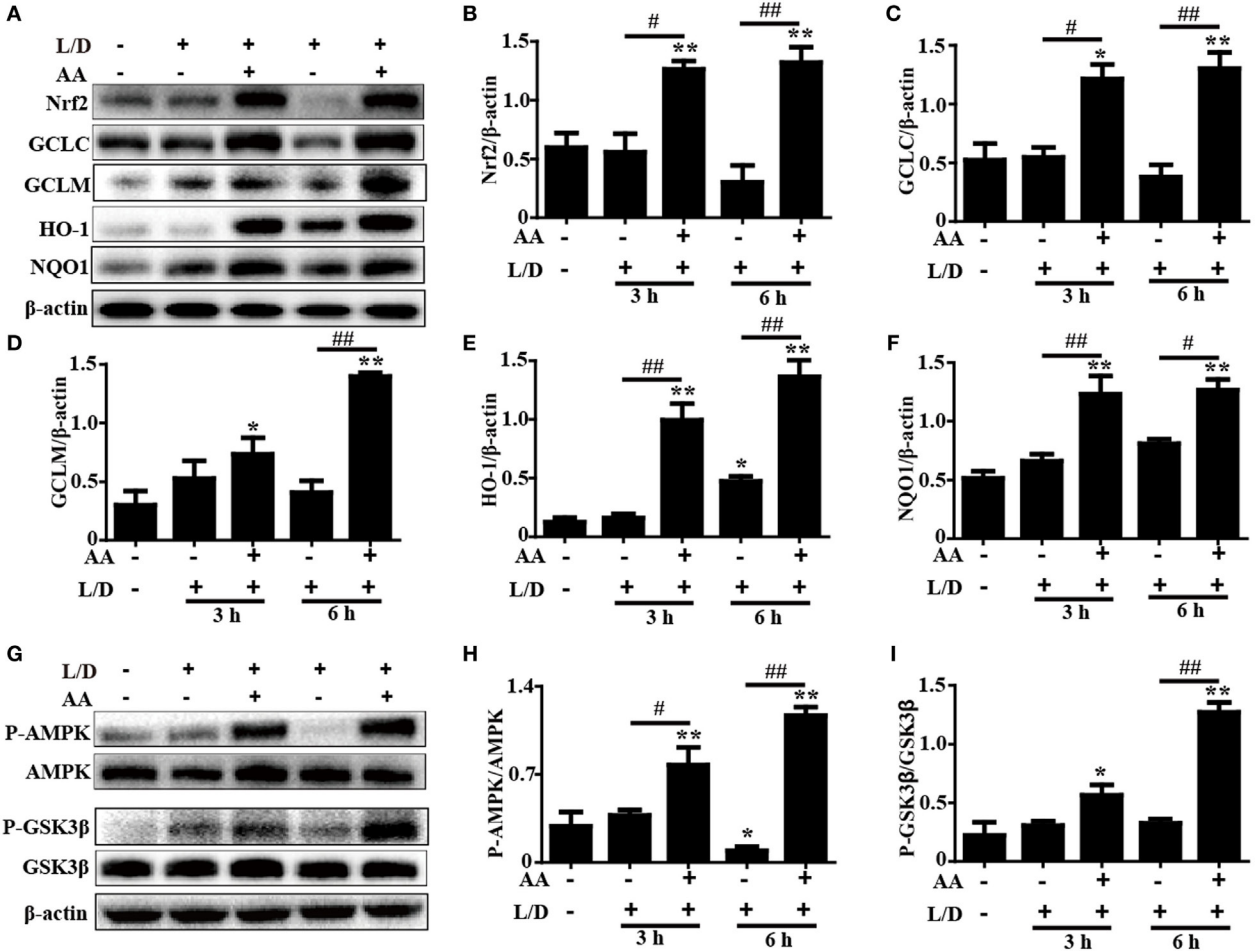
Effects of Asiatic acid (AA) treatment on the Nrf2 and AMP-activated protein kinase (AMPK)/GSK3β signaling pathways in L/D-induced fulminant hepatic failure. Liver tissues were collected from the mice at 3 or 6 h after L/D challenge, liver tissues were collected, and analyzed by Western blotting. **(A,G)** Effects of AA on Nrf2, glutamate-cysteine ligase catalytic (GCLC), glutamate-cysteine ligase modifier (GCLM), heme oxygenase-1 (HO-1), NQO1, P-AMPK, and P-GSK3β protein expression. **(B–F,H,I)** Quantification of relative protein expression was performed by densitometric analysis. β-actin was acted used as an internal control. Similar results were obtained from three independent experiments. All data are presented as means ± SEM (*n* = 5 in each group). *p < 0.05 and **p < 0.01 vs. control group; ^#^*p* < 0.05 and ^##^*p* < 0.01 vs. L/D group.

Increasing evidence indicates that the Nrf2-mediated signaling pathway is essential for the inhibition of oxidative stress and inflammatory responses in mice with L/D-induced FHF. Therefore, we examined whether the antioxidant and anti-inflammatory activities of AA were related to upregulation of the Nrf2-mediated signaling pathway. The inductions of GCLC, GCLM, HO-1, and NQO1, which are regulated by Nrf2 transcription, have been reported to be a key cellular protective mechanism against the inflammatory response or oxidative stress in various cell types (28, 29). As shown in Figures 5A–F, AA treatment remarkably enhanced Nrf2, GCLC, GCLM, HO-1, and NQO1 protein expression compared with the control group and the L/D-exposed group (*p* < 0.05 or *p* < 0.01).

Importantly, previous studies suggested that multiple signaling pathways modulated Nrf2 expression. Our previous study indicated that AA inhibited cellular damage and oxidative stress by regulating Nrf2 signaling dependent upon activation of the AKT and ERK signals in HepG2 cells (24). Accordingly, to investigate the protective mechanism of AA treatment on L/D-induced FHF, the ERK and PI3K/AKT activities were analyzed by Western blotting. Our results showed that AA treatment significantly inhibited L/D-induced phosphorylation of ERK, PI3K, and AKT, indicating that AA-mediated Nrf2 activation was unlikely to be associated with the ERK and PI3K/AKT signaling pathways *in vivo* (Figure S1 in Supplementary Material). However, AA treatment effectively increased AMPK and GSK3β phosphorylation compared with the L/D-exposed group (*p* < 0.05 or *p* < 0.01) (Figures 5G–I). Furthermore, we found that AA obviously induced AMPK and GSK3β phosphorylation and Nrf2 protein expression (*p* < 0.05 or *p* < 0.01) in HepG2 cells incubated with AA (6 µM) for different times, whereas these effects were efficiently blocked by compound C (CC, an AMPK inhibitor) (Figure S2 in Supplementary Material). Together, these investigations suggest that the ability of AA to suppress inflammation and oxidative stress in L/D-induced FHF may involve upregulation of the Nrf2-medicated signaling pathway *via* activation of AMPK/GSK3β *in vivo* and *in vitro*.

### AA-Induced PDCD4 Protein Expression Was Independent of Nrf2 Activation in HepG2 Cells

**Figure 6:**
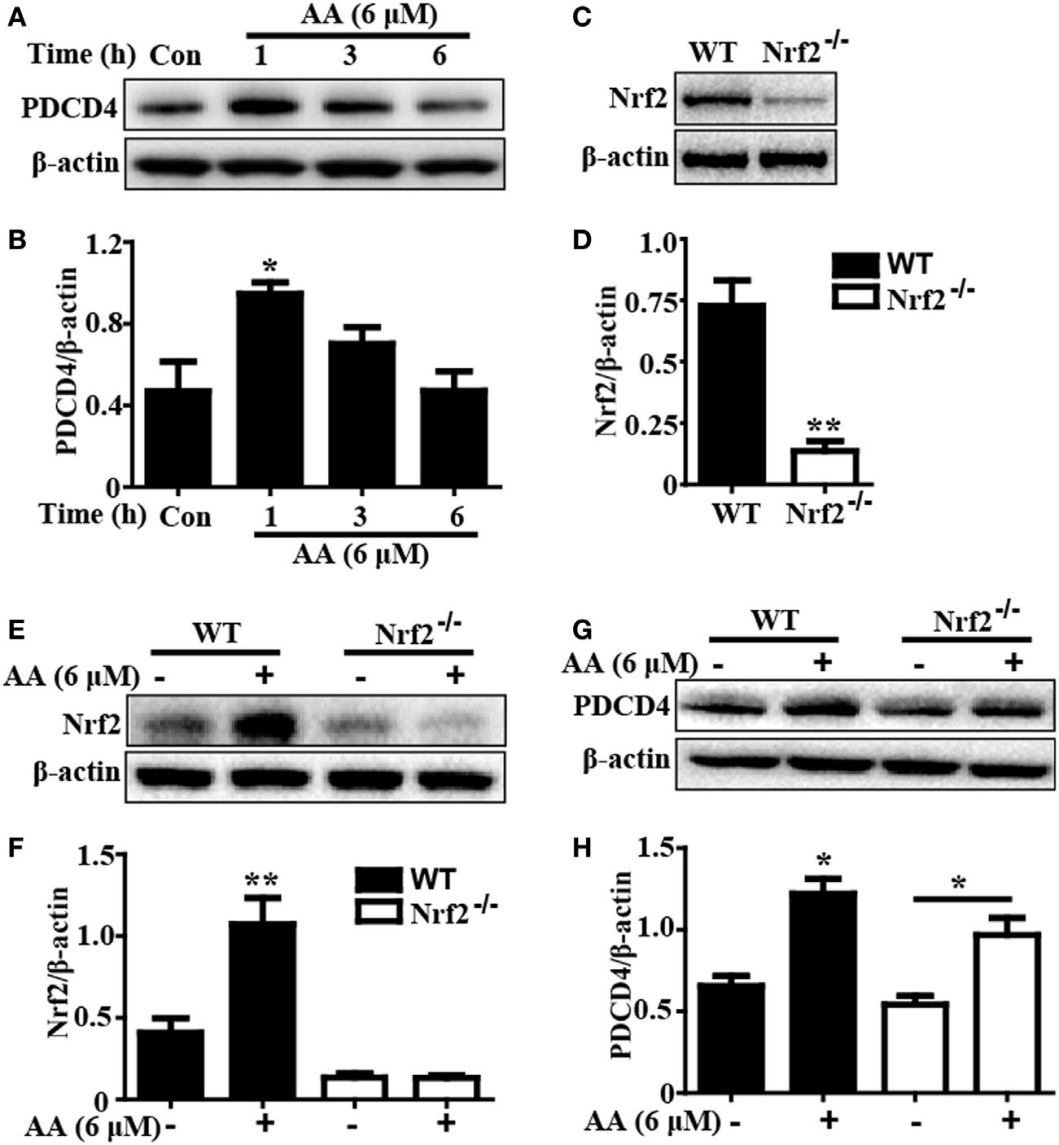
Asiatic acid (AA)-induced PDCD4 protein expression is independent upon Nrf2 activation in HepG2 cells. **(A)** HepG2 cells were pretreated with or without AA (6 µM) for 1, 3, or 6 h. Effects of AA on PDCD4 protein expression were measured by Western blotting analysis. Additionally, **(C,E)** WT and Nrf2^−/−^ HepG2 cells were pretreated with or without AA (6 µM) for 6 h. Effects of AA on Nrf2 protein expression were measured by Western blotting analysis. **(G)** WT and Nrf2^−/−^ HepG2 cells were pretreated with or without AA (6 µM) for 1 h. Effects of AA on PDCD4 protein expression were measured by Western blotting analysis. **(B,D,F,H)** Quantification of relative protein expression was performed by densitometric analysis and β-actin was acted used as an internal control. Similar results were obtained from three independent experiments. All data are presented as means ± SEM (*n* = 5 in each group). *p < 0.05 and **p < 0.01 vs. control group.

Based on the above outcomes, we evaluated whether the AA-induced PDCD4 expression was dependent of Nrf2 activation by using WT and Nrf2^−/−^ HepG2 cells. In the present study, after exposure of HepG2 cells to AA (6 µM) for different times, we found that AA exposure for 1 h obviously enhanced PDCD4 protein expression compared to the unexposed cells (*p* < 0.05) (Figures 6A,B). Nrf2 protein expression induced by AA was evidently inhibited in the Nrf2^−/−^ cells compared with the control cells, whereas AA-enhanced PDCD4 protein expression was not suppressed in the Nrf2^−/−^HepG2 cells (Figures 6C–H). These results suggested that AA-induced PDCD4 protein expression is independent of Nrf2 activation in HepG2 cells.

## Discussion

Inflammation and oxidative stress are considered strongly interrelated biological events that are involved in the pathogenesis of various diseases, including FHF (30–32). Emerging evidence suggests that LPS/d-GalN (L/D) give rise to hepatic injuries resulting from oxidative stress and inflammatory responses, which parallel those of viral hepatitis (33, 34). Moreover, GalN/LPS-induced liver failure is widely accepted as a key experimental liver injury model and has contributed to investigations of the mechanisms underlying clinical liver injury and screening of some efficient hepatoprotective agents (35). Accordingly, any approach that relieves inflammatory responses and oxidative stress *in vitro* and *in vivo* may conduce to the prevention or treatment of FHF. AA has been reported to possess various biological activities, including antioxidant and anti-inflammatory properties (19, 20). Therefore, the aim of this study was to investigate whether AA played anti-inflammatory and antioxidant roles in mice with L/D-induced FHF.

Under physiological conditions, ALT and AST are mostly present in liver cells; however, these enzymes are transferred through the cell membrane into the serum when liver cells are damaged (36), which implies serious impairment of liver functions. In our studies, AA treatment effectively decreased the ALT and AST levels in sera and increased survival rate in the L/D-induced mouse model. Conversely, previous studies have indicated that L/D-induced macrophages in FHF mice provoke the release of numerous inflammatory factors, including TNF-α, IL-6, and IL-1β, by liver cells, resulting in significant damage to the live structure and functions (37, 38). Our findings demonstrated that AA pretreatment efficiently restored damage of the hepatic architecture and reduced TNF-α, IL-6, and IL-1β secretion in mice with L/D-induced FHF. Recent studies have shown that ROS, such as the superoxide radical $\left(O_{2}^{-}\right)$, NO, and hydrogen peroxide (H_2_O_2_), are essential for the development of L/D-induced FHF (38, 39). Moreover, oxidative stress can increase the production of MPO and MDA formation to further result in liver tissue damage (40). In contrast, L/D-induced SOD and GSH depletion are involved in aggravating oxidative damage in mice with FHF (41). Our findings indicated that AA treatment remarkably inhibited L/D-induced ROS (${\text{O}}_{2}^{-}$, NO, and H_2_O_2_) formation and increased the GSH and SOD levels, suggesting that AA treatment significantly attenuated L/D-induced oxidative damage in mice with FHF. These investigations demonstrated that AA pretreatment relieved pathological changes, indicating potential for the use of AA in clinical applications for the prevention and treatment of liver injury *via* inhibition of inflammation and oxidative stress damage.

Based on the above outcomes, we investigated the protective mechanism of AA against L/D-induced inflammation and oxidative stress in mice with FHF. Increasing evidence has shown that L/D-induced activation of NF-κB and MAPK, which are associated with the regulation of cytokine generation (i.e., TNF-α, IL-1β, and IL-6 secretion), plays a pivotal role in mice with FHF (1, 42). Our results showed that AA treatment effectively inhibited L/D-induced JNK, ERK, P38, P65, and IκBα phosphorylation and blocked IκBα degradation. Moreover, PDCD4 downregulation was reported to promote LPS-stimulated secretion of proinflammatory mediators, and PDCD4 deficiency exacerbated the sensitivity of liver injury in L/D-induced mice by inducing MAPK and NF-κB pathway activation (8, 43). Our studies revealed that L/D exposure dramatically decreased PDCD4 protein expression, whereas this effect was significantly suppressed by AA pretreatment. Taken together, these results indicated that the inhibitory effects of AA pretreatment on L/D-induced inflammatory responses may be associated with suppression of NF-κB and MAPK *via* a mechanism that can partially be attributed to the induction of PDCD4 expression. Interestingly, previous reports discovered that inhibiting PDCD4 downregulation contributed to the induction of p21-dependent Nrf2 expression in HepG2 cells (44). In our study, AA-enhanced PDCD4 protein expression could not be suppressed in the Nrf2^−/−^ HepG2 cells, implying that AA-induced PDCD4 protein expression was independent of Nrf2 activation in HepG2 cells. Furthermore, previous studies have suggested that Nrf2, which is a multiple signaling pathway coordinator, possesses anti-inflammatory and antioxidant properties against acute and chronic diseases, including experimental liver injury, through regulation of the expression of various antioxidant genes, such as GCLC, GCLM, NQO1, and HO-1 (45, 46). In this work, AA significantly increased Nrf2, GCLC, GCLM, NQO1, and HO-1 protein expression, which was related to the anti-inflammatory and antioxidant activities exhibited in the mice with L/D-induced FHF. Importantly, recent reports revealed that Nrf2 transcription displayed hepatoprotective involvement in the activation of the AMPK/Akt/GSK3β signaling pathway in a liver injury model (19, 30). In our study, AA pretreatment obviously promoted AMPK and GSK3β phosphorylation in both HepG2 cells and mice with FHF. Although our previous research indicated that AA-activated Nrf2 signaling was majorly dependent upon AKT and ERK activation in HepG2 cells, AA treatment effectively inhibited the activation of PI3K/AKT and ERK induced by L/D in mice. Consequently, we speculated that this discrepancy resulted from the various cell types present in the livers of the mice, which differed from the results obtained using a single cell type. Finally, to examine whether AA-mediated Nrf2 transcription was associated with AMPK/GSK3β activation, HepG2 cells were exposed to the AMPK inhibitor compound C. Our investigations found that AA-induced Nrf2 activation was blunted by the AMPK inhibitor, suggesting that AA-enhanced Nrf2 induction might be dependent on the phosphorylation of AMPK and GSK3β *in vivo* and *in vitro*.

**Figure 7:**
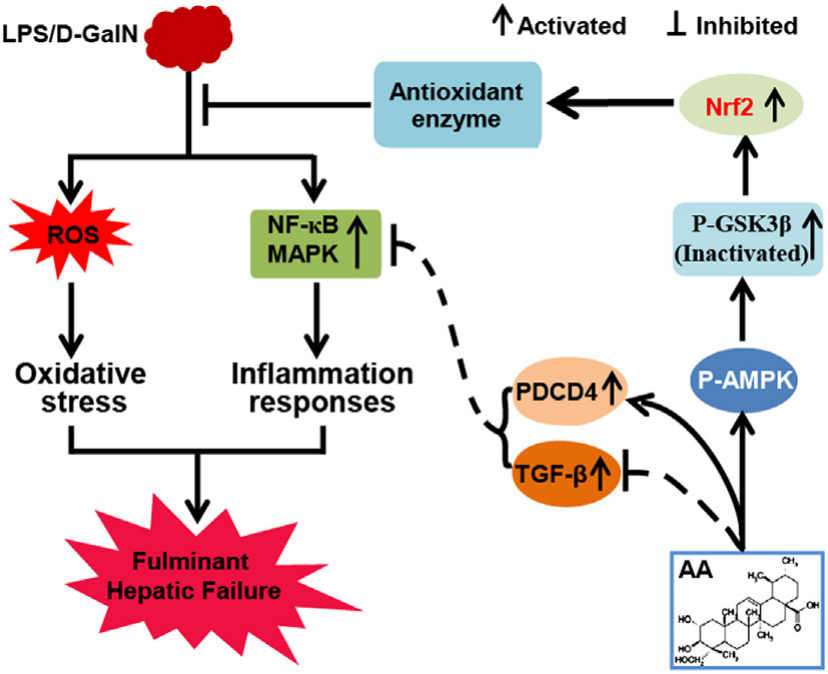
Asiatic acid (AA)-mediated Nrf2 signaling pathway protects lipopolysaccharide (LPS)/d-galactosamine (GalN)-induced fulminant hepatic failure (FHF) *via* inhibition of oxidative stress and inflammatory responses. (AA) can induce AMP-activated protein kinase and GSK3β activation, which contributes to upregulation of the Nrf2 signaling pathway and results in the expression of abundant antioxidant genes. Furthermore, AA not only reduces LPS/d-GalN-induced reactive oxygen species overproduction but also inhibits NF-κB and mitogen-activated protein kinase (MAPK) signaling pathway activation dependent upon induction of PDCD4 protein expression. These processes play significant roles in suppressing oxidative stress and inflammatory responses to alleviate FHF.

In conclusion, as shown in Figure 7, the investigations of this study suggested that AA played an essential role in liver protection by inhibiting inflammatory responses and oxidative stress. The underlying mechanisms may be closely associated with the inhibition of MAPK and NF-κB activation through the partial induction of PDCD4 and upregulation of Nrf2 in an AMPK/GSK3β pathway activation-dependent manner. Accordingly, the study provides beneficial evidence for the application of AA in protecting the liver from inflammatory and oxidative stress damage during FHF.

## Ethics Statement

All animal experiments were performed according to the guide for the Care and Use of Laboratory Animals, which was published by the US National Institute of Health.

## Author Contributions

HL wrote the paper and performed the experiments; ZQ performed the experiments; SW, HF, and XD analyzed the data; XC contributed to design the experiments.

## Conflict of Interest Statement

The authors declare that the research was conducted in the absence of any commercial or financial relationships that could be construed as a potential conflict of interest.

## Supplementary Material

Figure S1 Effects of asiatic acid treatment on PI3K/AKT signaling pathway in L/D-induced fulminant hepatic failure. Liver tissues were collected from the mice 3 or 6 h after L/D challenge and analyzed by Western blotting. **(A–C)** Quantification of relative expression of P-PI3K/PI3K and P-AKT/AKT were performed by densitometric analysis. Similar results were obtained from three independent experiments. All data are presented as means ± SEM (*n* = 5 in each group). ***p* < 0.01 vs. Control group; ^#^*p* < 0.05 and ^##^*p* < 0.01 vs. L/D group.

Figure S2 Effects of asiatic acid (AA) exposure on activation of the AMP-activated protein kinase (AMPK)/GSK3β/Nrf2 signaling pathways in HepG2 cells. **(A)** HepG2 cells were treated with AA (6 µM) for 1, 3, or 6 h. Western blotting analysis were used to explore phosphorylated AMPK and GSK3β expression as well as Nrf2 protein expression. **(E)** HepG2 cells were treated with compound C (CC, an AMPK inhibitor, 3 µM) for 18 h and then incubated with AA (6 µM) for another 6 h. **(B–D,F–H)** Quantification of relative protein expression was performed by densitometric analysis. β-actin was acted as an internal control. Similar results were obtained from three independent experiments. All data are presented as means ± SEM (*n* = 5 in each group). **p* < 0.05 and ***p* < 0.01 vs. control group.
