# Supplementary figures and images for: Asiatic Acid Exhibits Anti-inflammatory and Antioxidant Activities against Lipopolysaccharide and d-Galactosamine-Induced Fulminant Hepatic Failure
Source: Front Immunol. 2017 Jul 7;8:785. doi: 10.3389/fimmu.2017.00785 (PMC5500632; doi:10.3389/fimmu.2017.00785)

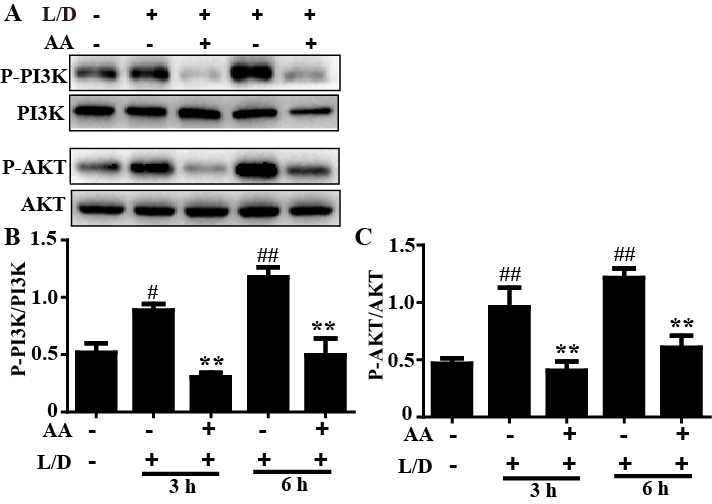

Supplement: Figure S1 — Effects of asiatic acid treatment on PI3K/AKT signaling pathway in L/D-induced fulminant hepatic failure. Liver tissues were collected from the mice 3 or 6 h after L/D challenge and analyzed by Western blotting. (A–C) Quantification of relative expression of P-PI3K/PI3K and P-AKT/AKT were performed by densitometric analysis. Similar results were obtained from three independent experiments. All data are presented as means ± SEM (n = 5 in each group). **p < 0.01 vs. Control group; #p < 0.05 and ##p < 0.01 vs. L/D group. [file image_1.tif]

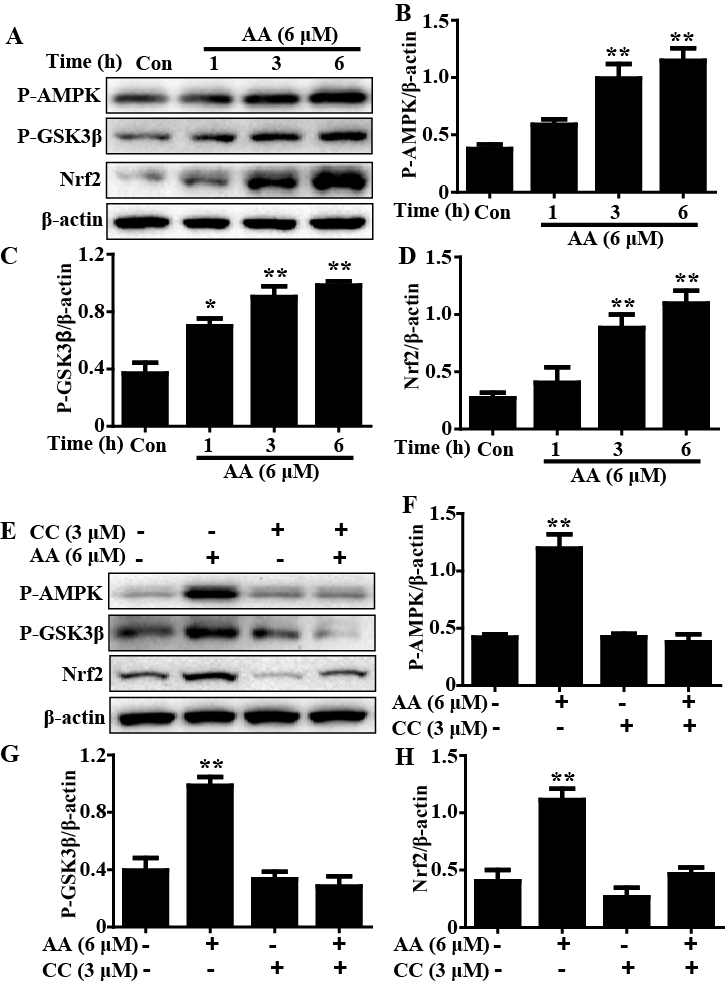

Supplement: Figure S2 — Effects of asiatic acid (AA) exposure on activation of the AMP-activated protein kinase (AMPK)/GSK3β/Nrf2 signaling pathways in HepG2 cells. (A) HepG2 cells were treated with AA (6 µM) for 1, 3, or 6 h. Western blotting analysis were used to explore phosphorylated AMPK and GSK3β expression as well as Nrf2 protein expression. (E) HepG2 cells were treated with compound C (CC, an AMPK inhibitor, 3 µM) for 18 h and then incubated with AA (6 µM) for another 6 h. (B–D,F–H) Quantification of relative protein expression was performed by densitometric analysis. β-actin was acted as an internal control. Similar results were obtained from three independent experiments. All data are presented as means ± SEM (n = 5 in each group). *p < 0.05 and **p < 0.01 vs. control group. [file image_2.tif]
